# An Image Encryption Algorithm Using Logistic Map with Plaintext-Related Parameter Values

**DOI:** 10.3390/e23111373

**Published:** 2021-10-20

**Authors:** Jakub Oravec, Lubos Ovsenik, Jan Papaj

**Affiliations:** Department of Electronics and Multimedia Communications, Technical University of Kosice, Nemcovej 32, 040 01 Kosice, Slovakia; lubos.ovsenik@tuke.sk (L.O.); jan.papaj@tuke.sk (J.P.)

**Keywords:** chaotic map, image encryption, logistic map, Lyapunov exponent, plaintext-related

## Abstract

This paper deals with a plaintext-related image encryption algorithm that modifies the parameter values used by the logistic map according to plain image pixel intensities. The parameter values are altered in a row-wise manner, which enables the usage of the same procedure also during the decryption. Furthermore, the parameter modification technique takes into account knowledge about the logistic map, its fixed points and possible periodic cycles. Since the resulting interval of parameter values achieves high positive values of Lyapunov exponents, the chaotic behavior of the logistic map should be most pronounced. These assumptions are verified by a set of experiments and the obtained numerical values are compared with those reported in relevant papers. It is found that the proposed design that uses a simpler, but well-studied, chaotic map with mitigated issues obtains results comparable with algorithms that use more complex chaotic systems. Moreover, the proposed solution is much faster than other approaches with a similar purpose.

## 1. Introduction

The extension of various communication networks and the increasing amount of transmitted data in the late 1970s caused the need for modern encryption algorithms. These algorithms were designed for operations with character strings, which allowed a wide spectrum of possible applications. For a long time, most of the research was related only to these algorithms and, since various alternatives were proposed much later, the first group of algorithms could be considered conventional algorithms.

The amount of applications for conventional encryption algorithms was further enlarged by introducing new modes of operation for them. However, in certain cases, even these tools could not make conventional encryption algorithms useful. Therefore, the research into dedicated encryption algorithms that could be utilized for some specific applications started.

The earliest image encryption algorithms from the late 1990s perceived the whole process of image encryption as the rearrangement of pixel intensities followed by some rather simple diffusion techniques [1,2]. These approaches exploited the properties of chaotic maps, which, in general, are dynamical systems that show unexpected and hardly predictable behavior [3]. After some time, researchers started to focus on the analysis of the image encryption algorithms and newly acquired knowledge led to significant changes in the design of image encryption algorithms. Probably the most important paper dealing with the analysis was published by Solak et al. in 2010 [4]. An attack described in [4] illustrated how several similar plain images could be used to reveal the architecture of the used image encryption algorithm or even parts of the used key. After the proposal of Solak’s attack, the majority of the newly designed image encryption algorithms employed more complex techniques that mitigated some vulnerabilities of previous approaches. At this point, some of the dedicated image encryption algorithms started to show better performance in certain applications than the conventional encryption algorithms such as the Advanced Encryption Standard (AES) [5]. These applications include the encryption of secret messages in image steganography systems [6,7,8] and securing medical images [9] or some biometric features [10]. The findings reported in [4] were later extended by Xie et al. in 2017 [11] and Preishuber et al. in 2018 [12].

**Figure 1:**
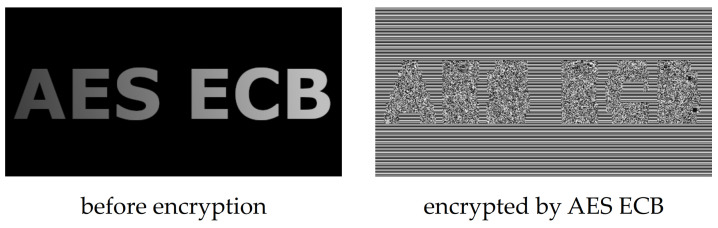
Effect of image encryption performed by AES in ECB mode.

Probably the largest issue with the usage of conventional encryption algorithms for image encryption is caused by the properties of uncompressed image data. While data stored in character strings are usually quite compact and individual characters are not related to each other, the number of image pixels could be large (to the extent that some of them are redundant) and their intensities are closely correlated [13,14]. This situation is shown in Figure 1, where a plain image with a resolution of 512 × 256 pixels and a color depth of 8 bits per pixel is encrypted by AES in its simplest mode of operation, being Electronic CodeBook (ECB) [15]. This example used password 0 × C9 0F DA A2 21 68 C2 34 C4 C6 62 8B 80 DC 1C D1, which was acquired from the first 128 bits of the binary expansion of number π.

Figure 1 shows the how AES works in ECB mode—it creates a codebook of encrypted data blocks that correspond to plain data blocks according to the used password. If a data block is present multiple times in the plain image, it will be reflected by one block of encrypted data on the same places in the encrypted image. This situation is visible mainly in monotonous areas of plain images, where the intensity changes between adjacent image pixels are very small or even absent.

The remedy for this problem could be the usage of another mode of operation for AES [15]. However, many other modes require more complex computations, which may raise the computational complexity of encryption. This is pronounced mainly on platforms that do not fully support hardware acceleration [16]. Another solution is the usage of a dedicated image encryption algorithm that is designed according to the specific properties of image data, such as the redundancy of image pixels and correlation of their intensities.

The newest generation of dedicated image encryption algorithms respond to the concerns raised by Solak et al. in [4] by so-called plaintext-related techniques. These include a broad spectrum of solutions; some of them will be briefly described in the following section. An important condition for every plaintext-related approach is that it has to utilize at least one step that depends on values obtained from plain images. In this case, the resulting encrypted image should be significantly different from images acquired from various plain images and the possibility of successful differential attacks (such as Solak’s attack) should be suppressed.

In this paper, we would like to propose a new approach to the plaintext-related image encryption. While the previous techniques focused on introducing plaintext-related steps mainly in the confusion and diffusion stages, the presented algorithm uses plain image pixel intensities to affect parameter values during the generation of a pseudo-random sequence. While some theoretical foundations were already laid out by Liu and Miao in 2016 [17] and some algorithms were even proposed, e.g., those by Chai et al. in 2020 [18] or Zheng and Hu in 2021 [19], all of these have significantly higher computational complexity than other plaintext-related techniques. The algorithm presented in this paper should provide a solution that has the benefits of a plaintext-related pseudo-random sequence combined with the favorable computational complexity of approaches that use a plaintext-related step during the confusion or diffusion stage.

There are also some other interesting papers dealing with the mentioned issues but, due to inappropriate choices of experimental images or a lack of reported numerical values, these approaches could not be effectively compared with other solutions. Some examples of these papers include [20,21,22,23,24].

The main contributions of this proposal are clearly described in a bullet-point list in Section 3.

The rest of the paper is organized as follows: Section 2 describes some of the recent work in the area of plaintext-related image encryption. Section 3 explains the proposed solution and also fundamental techniques that are used. Section 4 presents and discusses the experimental results. The last section, Section 5, concludes the paper with a brief overview of the properties of the proposed solution and some ideas for future work.

## 2. Related Work

One of the first image encryption algorithms that took into account Solak’s attack was designed by Kanso and Ghebleh in 2012 [25]. Their proposal changes the amount of chaotic map iterations according to plain image pixel intensities. While this design is beneficial against differential attacks, it increases the possibility of successful side-channel attacks as operations with brighter images require more time.

Another technique was presented by Fu et al. in 2013 [26]. In this case, the pixel intensities are converted to bits that are later rearranged by circular shifts with sizes determined by the intensities of previous image pixels. However, since there are only eight possible sizes of circular shifts corresponding to 256 possible pixel intensities, the same shift could be achieved by multiple pixel intensities.

A solution with a plaintext-related confusion stage was proposed by Zhang in 2014 [27]. The fact that plain image pixel intensities affect only the rearrangement of pixels together with the used architecture significantly decreases the performance of this approach, mainly for images with large monotonous areas.

Norouzi et al. presented a one-stage algorithm in 2014 [28] where the plain image pixel intensities are directly added to the processed intensity values. However, the drawbacks of this solution were reported by Zhang et al. already in 2014 [29] when Norouzi’s algorithm was declared as broken.

A plaintext-related algorithm designed by Murillo-Escobar et al. in 2015 [30] uses a sum of pixel intensities to modify some initial conditions of the utilized chaotic maps. There are two issues with this proposal—the same sum could be obtained from various images and this sum could not be computed from the encrypted image. Therefore, the decryption algorithm requires an additional parameter that is embedded into the encrypted image by means of steganography. The presence of this value is so obvious that the whole algorithm was broken by Fan et al. in 2018 [31].

Chai et al. proposed a technique employing a hash function in 2017 [32]. Since the hash digests from plain and encrypted images are significantly different, the decryption algorithm could not use the same key. This makes Chai’s algorithm asymmetric. Moreover, the usage of such complex tools as hash functions significantly increases the encryption and decryption times.

A similar solution with the hash functions was proposed by Wang et al. in 2018 [33]. In this case, even the authors admitted that the used architecture is complicated and the reported encryption speed of approx. 0.055 MB/s is quite low.

Since 2018, several authors have designed image encryption algorithms that use complex chaotic systems with five or more dimensions. While the computations of iterates take much more time, the performance is not always as good as in simpler and more finely tuned chaotic systems. The proposals with complex chaotic systems include two papers by Li et al. from 2018 and 2020 [34,35], where hash digests are computed multiple times and they are later used as inputs for other complex systems (Lorenz’s hyperchaotic system and piecewise linear chaotic map).

Sun’s algorithm from 2019 [36] is slowed down by a seven-dimensional chaotic system that is used to compute iterates only for three sequences. A solution by Chai et al. from 2020 [18] combines three simpler chaotic maps together with the Latin squares technique; however, the authors do not sufficiently analyze the potential drawbacks of the resulting system, such as fixed points or periodic cycles. An approach by Zhang and Han from 2021 [37] uses a six-dimensional system together with a technique of DNA coding, which results in very slow encryption speeds.

A proposal by Zheng and Hu from 2021 [19] utilizes Chen’s chaotic system and plain image pixel intensities to perturb the parameters of another chaotic system. This solution does not use hash functions and the properties of the resulting combined chaotic system are clearly described.

In our previous work, we focused on several topics regarding plaintext-related image encryption. A paper from 2018 [38] described how a two-dimensional chaotic map could be used for introducing dependencies between plain images and the steps of an image encryption algorithm. In a work from 2019 [39], an analysis of the one-way characteristics of the logistic map (LM) was given together with an algorithm that uses reported knowledge. A plaintext-related technique using the Mojette transform was presented in a paper from 2019 [40]. The most significant drawbacks of LM and their solutions were discussed in a publication from 2020 [41]. Our experience in the field of plaintext-related image encryption was used in a paper presented in 2021 [42] that describes a way to introduce relations between plain image pixel intensities and an encryption algorithm during the quantization of pseudo-random sequences.

## 3. Proposed Solution

The technique presented in this paper utilizes plain image pixel intensities for the modification of a parameter used by LM during the generation of one of the pseudo-random sequences. While similar techniques have been already investigated [17] and also experimentally tested [18,19], our proposal should be more effective—it should achieve comparable results with these, but with higher encryption speeds of older and simpler algorithms that utilize plaintext-related steps in the confusion or diffusion stage. The values of the commonly used numerical parameters of our proposal should be comparable with more complex approaches that use hash functions [32,33,34,35,36,37].

The main novelties of this proposal include:

- the usage of a novel plaintext-related parameter modification scheme for LM;
- the whole encryption/decryption scheme is symmetric—these operations are able to extract the required values from either plain or encrypted images;
- it takes into account the knowledge about LM—previously reported drawbacks such as fixed points or periodic cycles [39,41] are suppressed by careful choice of parameter value intervals and alternation of parameter values during the generation of pseudo-random sequences. This could be viewed as a novelty since it is not common even for new proposals.

**Figure 2:**
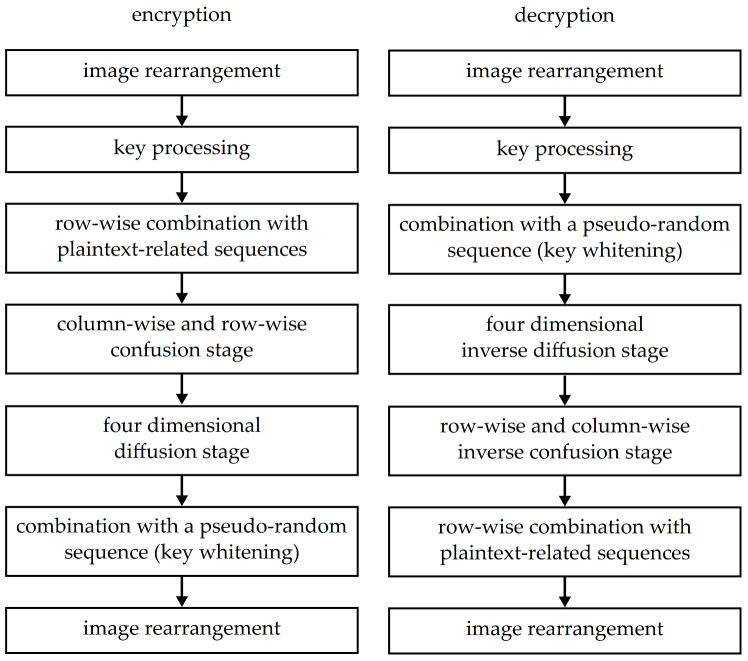
A block scheme describing stages of the proposed solution.

The presented approach can be applied on images with arbitrary resolution and color depths of 8 bits per pixel (grayscale images) or 24 bits per pixel (true color images). The key length is 128 bits and it is represented in a hexadecimal notation. A simplified block scheme of the proposed solution is shown in Figure 2.

Each stage presented in Figure 2 has its specific purpose. The image rearrangement stages prepare pixel intensities for processing (into two-dimensional matrices) or for encoding and saving the results (into images with color planes). The key processing stage divides the entered key into eight parts and converts them from hexadecimal notation to parameter values for the LM. Encryption continues by the plaintext-related stage, where the parameters of the generated sequence are changed according to the plain image pixel intensities. Then, the correlation of pixel intensities is suppressed by pixel rearrangement. New dependencies between their intensities are created during the diffusion stage. In the event that some of the pixel intensities are different, this stage spreads the differences across the whole image. A key whitening stage is especially important for providing better robustness against attacks, as it is the first stage that needs to be broken. In this stage, the processed image is combined with a pseudo-random sequence that depends on the used key. Since the sequence needs to be generated prior to the combination, this stage has to happen after the key processing stage, both during encryption and decryption. The whole concept of key whitening comes from conventional encryption algorithms; it is used also in AES [43].

Decryption uses a slightly different order of the mentioned stages. The first, second and the last stage are the same as during the encryption. The other decryption stages, which could be numbered 3 to 6, correspond to encryption stages, but their order is reversed—the combination with a pseudo-random sequence is followed by inverse diffusion and confusion stages and row-wise combination with plaintext-related sequences.

### 3.1. Logistic Map and Its Properties

LM can be considered an example of a chaotic system with a simple definition but rather complicated behavior [44]. LM is a one-dimensional map, so each iteration of the map generates one value, called an iterate. The computations of LM utilize one parameter r∈(0,4) and an initial value $x_{0}\in \left(0,1\right)$. Iterate values $x_{n}\in \left(0,1\right)$ are computed by (1):

(1) $$x_{n+1}=r\cdot x_{n}\left(1-x_{n}\right),$$

where n∈{1,2,3,⋯,N} is the sequential number of iterates and *N* is the total number of iterates.

The desired unpredictable behavior of the LM is achieved after some iterations that are used only to shift from the initial value $x_{0}$. These iterates that are not used for the generation of pseudo-random sequences belong to the so-called transient period. Its length is usually 1000 iterates [44].

**Figure 3:**
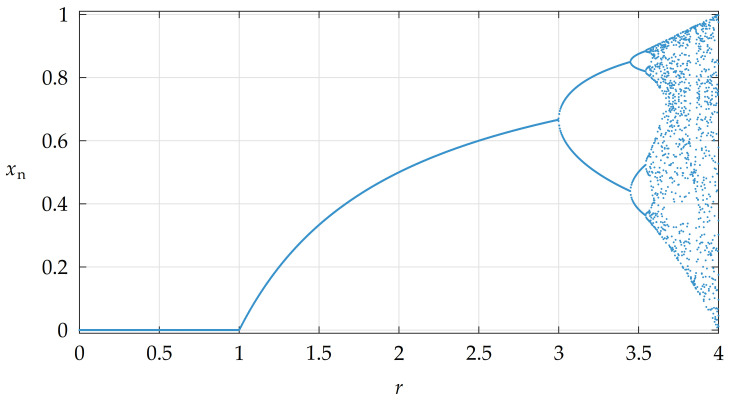
A bifurcation diagram for the LM.

The properties of the LM regarding various values of parameter *r* could be illustrated by a bifurcation diagram. An example of the bifurcation diagram, constructed from a sequence with $x_{0}=0.5$ and a transient period of 1000 iterates is shown in Figure 3.

The bifurcation diagram shows that the behavior of the LM is predictable until r∼3, when the first bifurcation occurs [3,44]. After several other bifurcations, the number of possible iterate value trajectories greatly increases and it becomes challenging to determine on which one the next iterate value would lie. There are still some areas with suppressed chaotic behavior, such as that around r∼3.85; however, the area close to r=4 displays the most unpredictable behavior. This could be illustrated also by a plot of estimated Lyapunov exponents (LEs) λ that quantify divergences between two trajectories with a small initial difference [3,41,44]. LEs for the LM could be estimated by (2) [41]:

(2) $$\lambda ∼\lim_{it\to \infty }\frac{1}{It}\sum_{it=0}^{It-1}\ln\left|r\left(1-2x_{it}\right)\right|,$$

where it=1,2,3,⋯,It is the sequential number of iterates computed for one parameter value *r*, It is the total number of these iterates, ln(a) is a natural logarithm of *a* and brackets |b| compute the absolute value of *b*.

**Figure 4:**
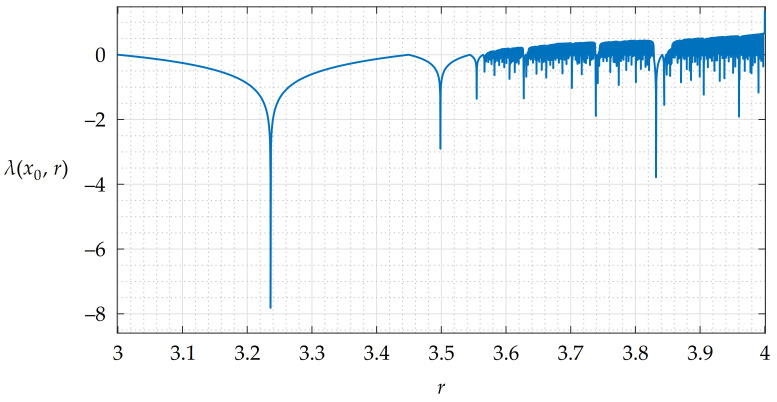
A plot of estimated LEs for the LM with r∈(3,4).

A plot of LEs estimated for a sequence of iterates generated by the LM (1) with $x_{0}=0.5$, parameter values of r∈(3,4), a transient period of 1000 iterates and It=1000 is shown in Figure 4. This plot has $10^{6}$ samples for the mentioned interval of *r*.

Positive values of λ indicate that the behavior of the LM at these values of *r* is considered chaotic. Negative spikes may be a sign that the LM has a periodic cycle or even a fixed point at corresponding values of *r* [41,44,45]. These situations are undesirable and, if they are not mitigated, image encryption algorithms could be susceptible to some of the attacks or they could be broken [46].

Locations of fixed points for the LM could be obtained by substitution and the solving of (1) [41,46]. If the interval of the used values of *r* for the LM is (0,4), the only fixed point is located at $\frac{1}{1-r}$. This fixed point can be suppressed by modification of *r* during the computation of new iterates. Multiple parameter values also suppress the occurrence of possible periodic cycles [41,45].

Another issue with the usage of the LM as a generator of pseudo-random sequences is dependencies between pairs of successive iterates and their unequal distribution. Both problems are caused by the nature of (1), which is an iterative function as $x_{n}=f\left(x_{n-1}\right)$. These two problems could be fixed by the usage of a suitable quantization technique [39,41].

While all mentioned issues with the LM have already published solutions, some of them are not very effective. In our previous work, we focused on the design of combined solutions that help to suppress several issues in one stage of the image encryption algorithm [41,42]. In this proposal, we would like to alter the parameter values of *r* according to the plain image pixel intensities. Moreover, the resulting values of *r* should achieve positive values of λ so that the generated sequences do not have any undesired statistical properties.

### 3.2. Encryption

The encryption algorithm uses a plain image *P* with arbitrary resolution and color depths of 8 or 24 bits per pixel. In addition to this, it needs also a 128-bit-long key *K*, inserted in a hexadecimal notation. The encryption produces an encrypted image *E*.

**Figure 5:**
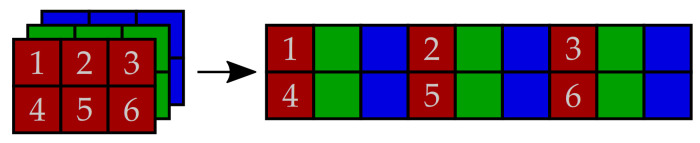
A rearrangement scheme for true color images.

**Step 1:** Image rearrangement. This step is used for the reshaping of both grayscale and true color images into a two-dimensional matrix $P^{\prime }$. The grayscale plain images are simply copied to matrix $P^{\prime }$. The color planes of true color images are decomposed into columns of pixels and these are rearranged as triplets consisting of columns from the red, green and blue color plane. This process is shown in Figure 5.

When a processed image is stored in matrix $P^{\prime }$, its width and height are passed to variables $w^{\prime }$ and *h*. The total number of image pixels is computed as $num_{px}=w^{\prime }\cdot h$ and the number of color planes is saved as $num_{cp}$.

**Step 2:** Key processing. Used key *K* is divided into eight parts $K_{1}$ to $K_{8}$. The hexadecimal characters from *K* are assigned to key parts according to (3):

(3) $$K_{i}\left(j\right)=K\left(2\cdot (i-1)+j\right),$$

where i=1,2,3,⋯,8 is the sequential number of key parts and j=1,2 is used as the sequential number of hexadecimal symbols.

The key parts $K_{1}$ to $K_{8}$ are then converted from hexadecimal to decimal notation with the usage of the big endian ordering scheme [47] and later used for the computation of parameter values $r_{1}$ to $r_{8}$ via (4):

(4) $$r_{i}=4-10^{-15}\left(\left(9-i\right)\cdot 256\cdot 65,536-K_{i}\right),$$

where constants of 256 and 65,536 represent the amount of possible plain image pixel intensities and key values.

**Figure 6:**
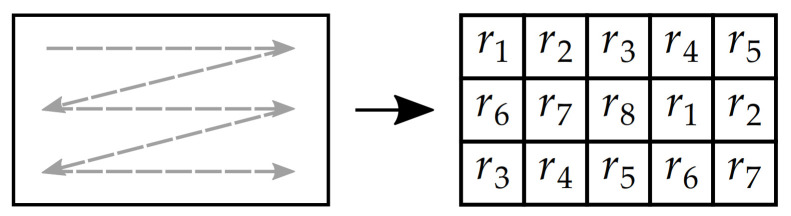
An illustration of a lookup table filled by row-major order.

**Step 3:** Row-wise combination with plaintext-related sequences. This step creates a lookup table LT with values of parameter *r* that are later modified by plain image pixel intensities and then used for combination with other pixel intensities. The lookup table LT has *h* rows and $w^{\prime }$ columns, being the same size as the matrix with processed image $P^{\prime }$. This step effectively doubles the memory consumption of the proposed solution, which is generally not an issue, but it greatly improves the speed of the whole algorithm. The lookup table LT is filled by repeating sequences of values $r_{1},r_{2},r_{3},\cdots ,r_{8}$ using a row-major order (at first, the values are passed to the top row from its left side to the right side, then to other rows) [47,48]. An example of a matrix filled by this technique is shown in Figure 6.

The values in the lookup table LT are then rearranged by two circular shifts that utilize two pseudo-random sequences $seq_{1}^{\prime }$ and $seq_{2}^{\prime }$. These are generated by the LM (1) with an initial value $x_{0}=0.5$ and a transient period of 1000 iterates. The key schedule used during the computation of all sequences, their length and maximal possible element values are shown in Table 1. The same parameter value patterns are used during the transient period and also after it.

Patterns of parameter values from Table 1 were not chosen with any specific intent. Operations with these parameter value patterns should result in similar values of numerical parameters for various plain images. The longest sequences, $seq_{3}$ and $seq_{6}$, use patterns from $r_{1}$ to $r_{8}$ and from $r_{8}$ to $r_{1}$, respectively. Sequences $seq_{4}$ and $seq_{5}$ used for the rearrangement of pixel intensities switch values in pairs of parameter values from $seq_{3}$ and $seq_{6}$. Finally, sequences $seq_{1}$ and $seq_{2}$ use patterns that start with either $r_{4}$ or $r_{5}$ and then increment or decrement their index by 4 (one half of the total parameter amount).

Elements of generated sequences $seq_{1}$ and $seq_{2}$ are quantized by (5) and resulting sequences are denoted as $seq_{1}^{\prime }$ and $seq_{2}^{\prime }$.

(5) $$seq_{i}^{\prime }=\left\lfloor Q_{i}\cdot \left(10^{4}\cdot seq_{i}\;\left(\mathrm{mod}\;1\right)\right)\right\rfloor .$$

It should be noted that the quantization by (5) removes the first four decimal places of iterates. This is helpful for obtaining a balanced distribution of element values and also for the suppression of dependencies between successive sequence elements [39,41].

**Figure 7:**
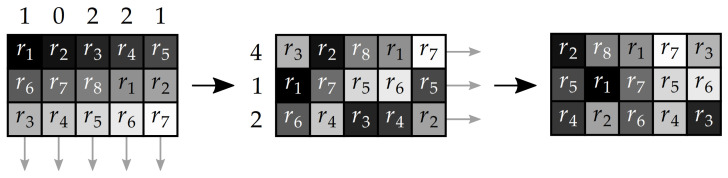
A matrix rearrangement technique.

The first group of circular shifts rearranges the parameter values in the individual columns of the lookup table LT. The shift sizes are determined by values of sequence $seq_{1}^{\prime }$. Then, the second group of shifts is done in the rows of LT, with the sizes of shifts set by sequence $seq_{2}^{\prime }$. An illustration of the described rearrangement scheme is displayed in Figure 7.

Then, a sequence $seq_{3}$ is generated by LM (1), but this time in a different way. It uses $x_{0}=0.5$, but after its transient period of 1000 iterates, only one iterate $x_{1001}$ is stored. This iterate is used as a starting point for multiple sequences utilized for individual rows of the matrix with processed image $P^{\prime }$.

Now, this step works individually with each row of pixel intensities from matrix $P^{\prime }$. The rows are scanned from the top to the bottom, with their indexes being l=1,2,3,⋯,h. For each of these rows, a sequence $seq_{plr}$ with $w^{\prime }$ elements is generated by the LM (1) without any other transient periods and with an initial value of $x_{1001}$. LM uses parameter values from row *l* of lookup table LT that are modified by plain image pixel intensities from row l−1 of $P^{\prime }$ by (6):

(6) $$LT\left(l,:\right)=LT\left(l,:\right)+10^{-15}\cdot 65,536\cdot P^{\prime }\left(l-1,:\right),$$

where the colon : stands for all indexes in a row of image pixels, the constant of 65,536 represents the amount of possible key values and index l−1 is substituted with *h* for the first row of $P^{\prime }\left(1,:\right)$.

The resulting modified parameter values *r* in lookup table LT belong to interval $\langle 3.999999865833743,4-10^{-15}\rangle$ in a double precision data type [49]. Since the minimal value of LEs estimated with $x_{0}=0.5$ for this interval with a transient period of 1000 iterates and It=1000 is still positive at approx. 0.6645 for *r* = 3.9999999629572112, the sequences generated with these parameter values are considered chaotic [3,41,44].

The sequence $seq_{plr}$ generated with plaintext-related parameters from lookup table LT is quantized by (5), stored as $seq_{plr}^{\prime }$ and then it is combined with the currently scanned row of pixel intensities in $P^{\prime }$ by (7):

(7) $$P^{\prime }\left(l,:\right)=P^{\prime }\left(l,:\right)⊕seq_{plr}^{\prime },$$

where ⊕ represents an operation of binary eXclusive OR (XOR) [50,51].

The procedures dealing with the lookup table LT—modification of values according to the intensities of $P^{\prime }$, generation and quantization of the sequences and their combination with processed image $P^{\prime }$—are repeated for all other rows. The scanning order from the top to the bottom of the $P^{\prime }$ is important, as it could be reversed during decryption (l=h,h−1,h−2,⋯,1). Hence, the decryption algorithm is able to obtain the required pixel intensities from $P^{\prime }\left(l-1,:\right)$ that affect the parameter values in LT.

**Step 4:** Column-wise and row-wise confusion stage. Any traces of pixel intensity correlation that could be left in matrix $P^{\prime }$ after Step 3 are suppressed by rearrangement of its pixels. This step is done similarly as the shuffling of parameter values $r_{1}$ to $r_{8}$ in the lookup table LT. At first, two sequences $seq_{4}$ and $seq_{5}$ are generated by the LM (1) with initial values $x_{0}=0.5$ and other parameters given by Table 1. These sequences are then quantized by (5) and stored as $seq_{4}^{\prime }$ and $seq_{5}^{\prime }$.

After this, the circular shifts in the individual columns and individual rows of matrix $P^{\prime }$ are done. The sizes of the shifts are determined by the element values of sequences $seq_{4}^{\prime }$ and $seq_{5}^{\prime }$. This technique is shown also in Figure 7.

**Step 5:** Four-dimensional diffusion stage. This step introduces dependencies between pixel intensities from matrix $P^{\prime }$, which are useful when two similar plain images are encrypted. The dependencies are created in four directions so all pixel intensities of $P^{\prime }$ are affected even by small differences between plain images. During each of the four scans, the actually processed vector of pixel intensities from $P^{\prime }$ is combined with two other vectors—one is added by modulo 256 addition and the second one is XORed with the actually processed vector. Indexes of all vectors used during the four scanning directions are described in Table 2.

If row index l+1 or column index k+1 is greater than *h* or $w^{\prime }$, a value of 1 is used instead. Furthermore, if indexes l−1 or k−1 are less than 1, values of *h* or $w^{\prime }$ are utilized.

**Step 6:** Combination with a pseudo-random sequence. This step helps to protect all previous steps as any successful attacks need to break this step at first. A sequence $seq_{6}$ is generated by the LM (1) with an initial value $x_{0}=0.5$, a transient period of 1000 iterates and other parameters given by Table 1. This sequence is quantized by (5), stored in a variable $seq_{6}^{\prime }$ and later rearranged to a matrix $seq_{6m}^{\prime }$ with *h* rows and $w^{\prime }$ columns by the row-major order shown in Figure 6. The matrix $seq_{6m}^{\prime }$ is then combined with matrix $P^{\prime }$ by (8):

(8) $$P^{\prime }=P^{\prime }⊕seq_{6m}^{\prime }.$$

**Step 7:** Image rearrangement. In this step, the matrix with processed image pixel intensities $P^{\prime }$ is transferred into encrypted image *E*, which is the sole output of the encryption algorithm. The rearrangement scheme is inverse to that presented in Step 1—if the value of $num_{cp}$ points out that the plain image *P* was true color, three color planes are reconstructed from triplets of columns from $P^{\prime }$. Otherwise, if the plain image *P* was grayscale, it is directly copied from matrix $P^{\prime }$ to image *E*.

### 3.3. Decryption

The decryption algorithm uses an encrypted image *E* and 128-bit-long key *K* to produce a decrypted image *D*. As already shown in Figure 2, the decryption algorithm stages are almost the same as those used during encryption; however, the order of some is reversed. The first two steps are the same. The third decryption step is an inverse of the sixth encryption step—the processed image matrix is combined with a sequence generated by the LM (the sequence that is not plaintext-related). After this, next step of the decryption algorithm removes dependencies created during the four-dimensional diffusion stage. At this step, the order of scanning directions is reversed and the signs for addition modulo 256 are changed from “+” to “−” and vice versa.

After this, the pixel intensities are rearranged back in the fifth decryption step by two groups of circular shifts. Their order is reversed—the first group of shifts takes place in rows of matrix $P^{\prime }$ and the second group deals with shifts in the columns of $P^{\prime }$. The sizes of shifts determined by sequences $seq_{5}^{\prime }$ and $seq_{4}^{\prime }$ are multiplied by a factor of −1. Then, in the sixth decryption step, the plaintext-related sequence is generated, processed and used for combination with $P^{\prime }$. Since the operation is row-wise and it could start at the bottom of the image and continue to its top, the decryption algorithm is able to revert the effects of the third encryption step without any additional information.

The decrypted image *D* is created from the matrix $P^{\prime }$ in the last step of the decryption algorithm by the same procedure as in the last encryption step.

## 4. Experimental Results

**Figure 8:**
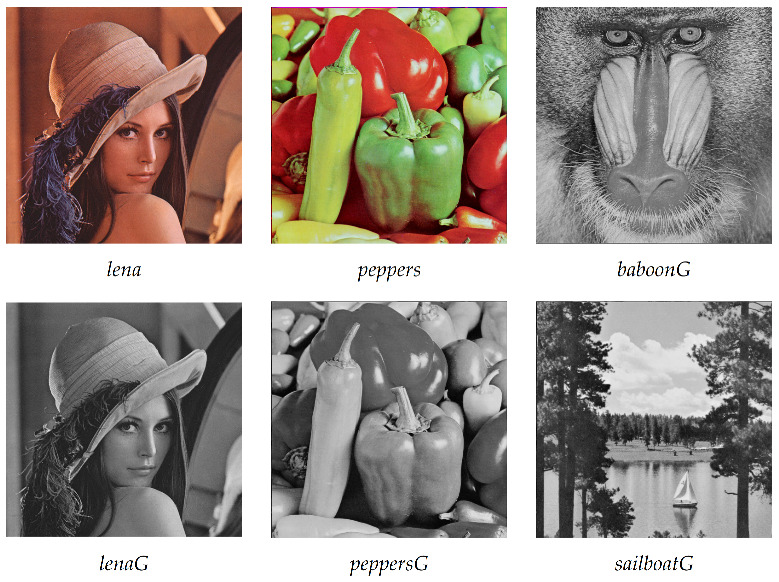
A set of experimental images.

Experiments with the proposed solution were performed on a PC with 2.5 GHz CPU, 12 GBs of RAM running MATLAB R2015a on Windows 10 OS. A set of images from the USC-SIPI database [52] used for the experiments is shown in Figure 8. All these images have a resolution of 512 × 512 pixels. Images lena and peppers have color depths of 24 bits per pixel; other images have color depths of 8 bits per pixel. Utilized keys are included in Table 3. The value of key $K_{1}$ was obtained from the first 128 bits of the binary expansion of number π. Please note the minimal difference between keys $K_{1}$ and $K_{2}$.

### 4.1. Key Space Size and Key Sensitivity

The proposed image encryption algorithm utilizes 128-bit-long keys. Therefore, the size of key space is $2^{128}$. Considering that the decryption of a grayscale image with a resolution of 512 × 512 pixels takes approx. 140 ms (see Section 4.6 for details), the brute force attack requires approx. $1.5106\times 10^{30}$ years. Hence, the proposed image encryption algorithm can be considered robust enough against brute force attacks.

**Figure 9:**
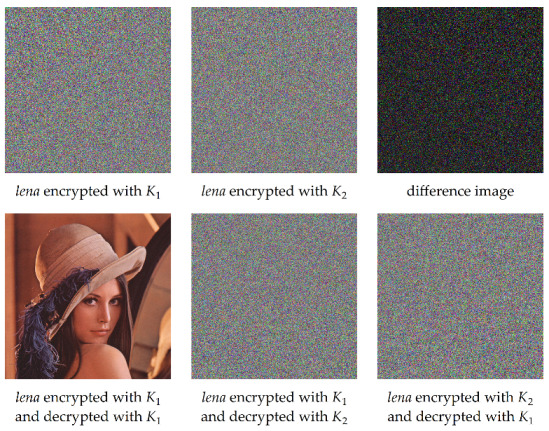
An illustration of key sensitivity of the proposed solution.

The effects caused by the usage of incorrect keys are shown in Figure 9. Even the smallest possible difference between keys $K_{1}$ and $K_{2}$ results in major differences between two encrypted or decrypted images. This means that the proposed image encryption algorithm is sensitive to the used keys.

### 4.2. Robustness against Image Modification

**Figure 10:**
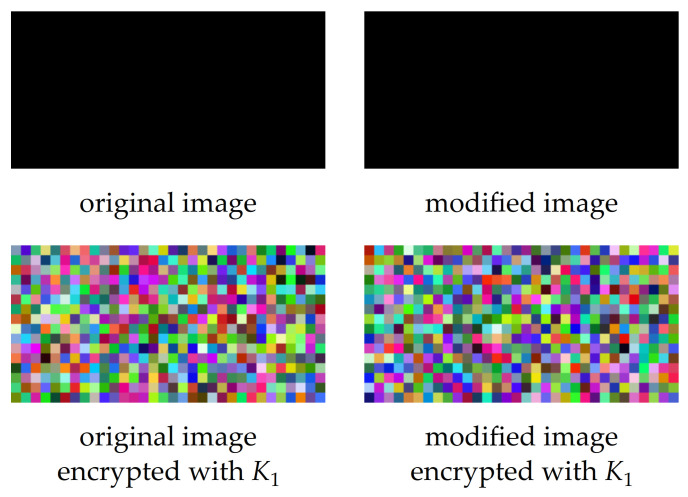
Effect of even slight modification on encrypted images.

The proposed image encryption algorithm was designed to be sensitive to even slight differences between plain images. In the event that two plain images differ in the intensities of one of more image pixels, the encryption by the proposed image encryption algorithm leads to significantly different encrypted images. An example of this feature is shown in Figure 10, where two similar plain images with a resolution of 32 × 16 pixels and color depth of 24 bits per pixel were encrypted with key $K_{1}$. All pixel intensities of the original plain image were equal to zero; the modified plain image had one pixel with an intensity level of 1 in the top left corner of the red color plane.

The mentioned property of the proposed image encryption algorithm means that it is not robust to any modification of plain or encrypted images. Each change would affect the resulting image.

### 4.3. Statistical Properties of the Plaintext-Related Sequence

Since one of the sequences generated by the LM (1) is modified by plain image pixel intensities, we supposed that it might be interesting to investigate the statistical properties of this sequence. For this purpose, the NIST 800-22 test suite [53] was utilized. The same set of statistical tests was used during the AES candidate selection process.

The NIST 800-22 suite runs 15 statistical tests over a set of binary sequences. In our case, we followed the recommendations given in [53] and used 100 sequences with a length of $10^{6}$ bits. Therefore, the required length of the plaintext-related sequence was $10^{8}$ bits. This sequence was obtained from the encryption of a zero-intensity image (intensities of all pixels are equal to 0) with a resolution of 3000 × 2000 pixels and color depth of 24 bits per pixel. The first $10^{8}$ element values of sequences $seq_{plr}^{\prime }$ were converted to binary notation by the big endian ordering scheme [47] and stored in a vector that was later tested by the NIST 800-22 test suite. Encryption used key $K_{3}$, which is a zero key (all elements are 0)—this combination of plain image and key is practically the worst-case scenario for the image encryption algorithm as both the plain image and key are very monotonous.

The results in Table 4 show that the analyzed sequence displays suitable statistical properties even after its modification according to the plain image pixel intensities. The analyzed sequence passed all 15 tests, and borderline results were obtained in the runs, overlapping template matching and approximate entropy tests.

### 4.4. Properties Regarding Statistical Attacks

**Figure 11:**
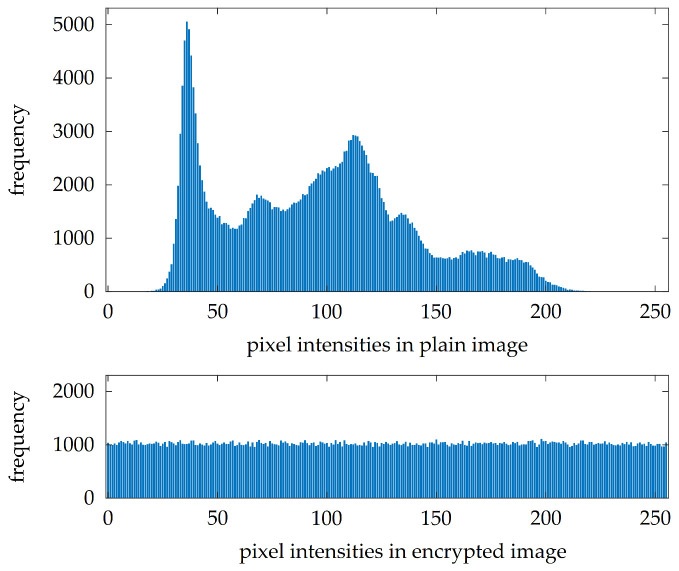
A comparison of histograms of plain and encrypted images.

The robustness of encrypted images against statistical attacks could be evaluated by several measures. The first of them is the suppression of peaks in the histograms of encrypted images. A histogram comparison for plain image lenaG and its version encrypted with key $K_{1}$ is shown in Figure 11. It is clearly visible that the proposed image encryption algorithm flattens the histogram; therefore, it is more difficult to extract some useful statistical information from the encrypted image.

Histogram comparison could be done also in an objective way by computing values of histogram variance var by (9). Higher values of var mean that the histogram has significant peaks and smaller values of var point out that it is more balanced. Values of var for the set of experimental images and keys are included in the third column of Table 5.

(9) $$var=\frac{1}{2^{2\cdot L}}\sum_{i=1}^{2^{L}}\sum_{j=1}^{2^{L}}\frac{{\left(hg(i)-hg(j)\right)}^{2}}{2}\;\left[-\right],$$

where *L* is the color depth of a color plane or grayscale image, *i* and *j* are histogram bin indexes and hg denotes a histogram of the analyzed image.

**Figure 12:**
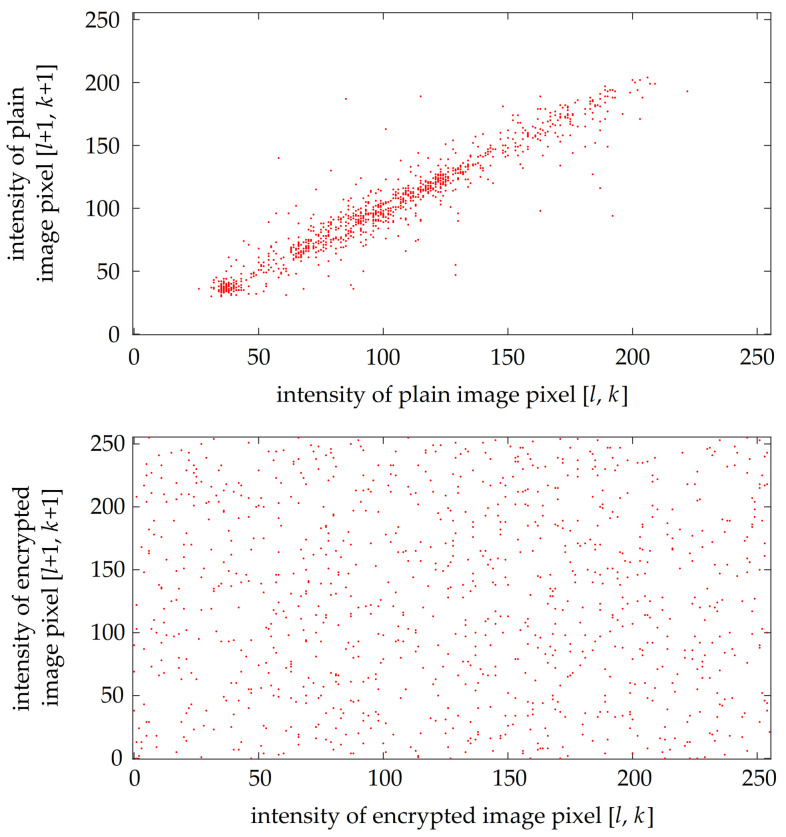
A comparison of correlation diagrams for plain and encrypted images.

Another means of evaluating the robustness against statistical attacks is through correlation diagrams. These plots generally use a set of randomly chosen 1000 pixel pairs. Each pair contains the intensities of two pixels that are adjacent either horizontally, vertically or diagonally. Then, the two intensities from each pixel pair are used as coordinates on two axes. If the intensities are close to each other, the resulting plotted point is close to line y=x. Otherwise, if the intensities differ a lot, the plotted point could be located anywhere in the plot. An example of a correlation diagram for 1000 randomly chosen diagonally adjacent pixel pairs from plain image lenaG and its version encrypted with key $K_{1}$ is shown in Figure 12. It can be clearly seen that the encryption breaks the correlation between the intensities of adjacent pixels.

The correlation between two adjacent pixel intensities could be assessed also by an objective measure—the value of correlation coefficients ρ. These are calculated separately for each color plane and in three different directions—horizontally ($\rho_{h}$), vertically ($\rho_{v}$) and diagonally ($\rho_{d}$)—by (10). Resulting values of $\rho_{h}$, $\rho_{v}$ and $\rho_{d}$ obtained from computations with 1000 randomly chosen pixel pairs are presented in columns 4 to 6 of Table 5. The interval of ρ is 〈−1,1〉, and lower absolute values of ρ mean that the image pixel intensities are less correlated.

(10) $$\rho =\frac{\sum_{pp=1}^{num_{pp}}\left(in_{1}\left(pp\right)-\bar{in_{1}}\right)\cdot \left(in_{2}\left(pp\right)-\bar{in_{2}}\right)}{\sqrt{\sum_{pp=1}^{num_{pp}}{\left(in_{1}\left(pp\right)-\bar{in_{1}}\right)}^{2}\cdot \sum_{pp=1}^{num_{pp}}{\left(in_{2}\left(pp\right)-\bar{in_{2}}\right)}^{2}}}\;\left[-\right],$$

where $pp=1,2,3,\cdots ,num_{pp}$ is the pixel pair index, $num_{pp}$ denotes the number of pixel pairs, sequences $in_{1}$ and $in_{2}$ store intensities from the pixel pairs and $\bar{in_{1}}$ stands for the arithmetic mean of sequence $in_{1}$.

The next objective measure is a value of entropy *H*, which is computed separately for each color plane of the analyzed image by (11). Values obtained for the sets of experimental images and keys are included in the seventh column of Table 5. The theoretical boundary of *H* is the same as the color depth of the investigated color plane or grayscale image. The higher values of entropy mean that the color plane or grayscale image is closer to an ideal source of random information [54].

(11) $$H=-\sum_{in=0}^{2^{L}-1}p\left(in\right)\cdot \log_{2}\left(p(in)\right)\;\left[\mathrm{bits}/\mathrm{px}\right],$$

where in is a vector of image pixel intensities and p(in) stands for the probability of the occurrence of a pixel with intensity in.

### 4.5. Properties Regarding Differential Attacks

In general, differential attacks use a pair of similar plain images $P_{1}$ and $P_{2}$, encrypt them with the same key and compare the resulting encrypted images $E_{1}$ and $E_{2}$. The difference between plain images is usually minimal—one pixel intensity is either incremented or decremented. There are two numerical parameters that assess robustness against differential attacks—the Number of Pixel Change Ratio (NPCR) and the Unified Average Changing Intensity (UACI). Since the location of the difference between plain images $P_{1}$ and $P_{2}$ could affect the resulting values of NPCR and UACI, both of these measures are computed as arithmetic means of 100 measurements with different locations of the pixel intensity difference.

NPCR sums up the amount of differences between two encrypted images $E_{1}$ and $E_{2}$. Its values are computed separately for each color plane or grayscale image by (12):

(12) $$\begin{array}{cc} NPCR= & \frac{100}{h\cdot w}\sum_{l=1}^{h}\sum_{k=1}^{w}Diff\left(l,k\right)\;\left[\%\right]\\ Diff(l,k)= & \left\{ \begin{array}{cc} 0 & \mathrm{if}\;E_{1}\left(l,k\right)=E_{2}\left(l,k\right)\\ 1 & \mathrm{if}\;E_{1}\left(l,k\right)\neq E_{2}\left(l,k\right) \end{array}\right. \end{array},$$

where *h* and *w* are the height and width of images, and *l* and *k* are line and column indexes.

On the other hand, UACI also takes into account the sizes of pixel intensity differences. The values of UACI are calculated separately for each color plane or grayscale image via (13):

(13) $$UACI=\frac{100}{h\cdot w}\sum_{l=1}^{h}\sum_{k=1}^{w}\frac{|E_{1}\left(l,k\right)-E_{2}\left(l,k\right)|}{2^{L}-1}\;\left[\%\right],$$

where brackets |a| represent the absolute value of number *a*.

Computed values of NPCR and UACI are presented in the last two columns of Table 5. Please note that the values for plain images are not included as the computation of NPCR and UACI could be done only for encrypted images.

The robustness of the analyzed image encryption algorithm against differential attacks can be considered sufficient if the computed values of the NPCR and UACI fall into intervals of expected values proposed by Wu et al. [55]. For images with a resolution of 512 × 512 pixels and a color depth of 8 bits per pixel, the intervals of 〈99.6094%,100%) for NPCR and (33.3115%,33.6156%〉 for UACI mean that the analyzed image encryption algorithm is robust against differential attacks in 999 of 1000 cases.

### 4.6. Measurement of Computational Complexity

The computational complexity of image encryption algorithms could be estimated by two methods. The first one examines the complexity of each operation used to encrypt or decrypt images by means of a big O notation [47]. However, it is challenging to apply this approach on image encryption algorithms as it is difficult to break complex operations such as matrix rearrangement, circular shifts or conversion from hexadecimal to binary notation into fundamental ones such as addition, subtraction or multiplication.

Therefore, image encryption algorithms are usually compared by the arithmetic means of repeated measurements of encryption times $t_{enc}$ and decryption times $t_{dec}$. The most common size of a measurement set is 100 times for encryption and 100 times for decryption. The effects of different image resolutions and color depths could be mitigated by the computation of encryption speeds $v_{enc}$ and decryption speeds $v_{dec}$ by (14):

(14) $$v_{oper}=\frac{h\cdot w\cdot num_{cp}}{2^{20}\cdot t_{oper}}\;\left[\mathrm{MB}/s\right],$$

where *h*, *w* and $num_{cp}$ denote the height, width and number of color planes of the analyzed image, the constant of $2^{20}$ stands for the number of bytes in a megabyte and $t_{oper}$ is the measured encryption time ($t_{enc}$) or measured decryption time ($t_{dec}$). The measurements use seconds as units.

One of the most important hardware parameters, the processor clock frequency, is taken into account in calculations of the numbers of processor cycles necessary for an encryption ($cyc_{enc}$) or a decryption ($cyc_{dec}$) of one byte by (15):

(15) $$cyc_{oper}=\frac{f_{CPU}\cdot t_{oper}}{h\cdot w\cdot num_{cp}}\;\left[\mathrm{cycles}/B\right],$$

where $f_{CPU}$ is the processor clock frequency in Hertz.

The arithmetic means of 100 repeated measurements of encryption and decryption times are presented as $t_{enc}$ and $t_{dec}$ in Table 6. These values were used for the computation of encryption speeds $v_{enc}$, decryption speeds $v_{dec}$ and also the numbers of processor cycles necessary for an encryption $cyc_{enc}$ or a decryption $cyc_{dec}$ of one byte. All these values are included in Table 6.

### 4.7. Discussion

The values presented in Table 5 lead to several conclusions. Encryption of all combinations of plain images and keys resulted in a significant decrease in histogram variance var. This fact is valid for both true color and grayscale images. The differences between the individual color planes of true color images are barely noticeable and they do not form any kind of pattern.

Correlation coefficients $\rho_{h}$, $\rho_{v}$ and $\rho_{d}$ are also visibly decreased after the encryption. None of the used keys obtains significantly better values. The results are balanced also for different plain images. This means that the proposed image encryption algorithm obtains very good results even after encryption with a monotonous key such as $K_{3}$.

Computed values of entropy *H* are close to the theoretical boundary of 8 bits per pixel. All presented values fall into a rather small interval of 〈7.9992,7.9994〉.

All values of NPCR and UACI belong to intervals of expected values for images with this resolution and color depth [55]. Different color planes of true color images have similar values of NPCR or UACI. The presented values are not affected by the value of the used key since results for all three experimental keys are very similar.

Comparison of encryption and decryption times $t_{enc}$ and $t_{dec}$ from Table 6 shows that they are slightly different—the decryption seems to be faster. The maximal differences between $t_{enc}$ and $t_{dec}$ for true color and grayscale images are similar at approx. 5.5 ms and approx. 6 ms, which means that this difference is not produced by the amount of processed data. This difference is caused by the reversed order of certain stages during decryption (see Figure 2 for details) as the processed image does not need to be saved to a matrix and loaded from it as many times as during encryption.

The encryption speeds $v_{enc}$ and decryption speeds $v_{dec}$ demonstrate that the speed of the proposed solution decreases with the increasing amount of processed data. This is caused by the linear complexity of the plaintext-related stage—the more sequence elements are generated and modified according to the plain image, the more time it takes. While this stage does not use hash functions or any similar tools, it is still the most complex among other stages used in the proposed algorithm. This issue is visible also from the values of measures $cyc_{enc}$ and $cyc_{dec}$.

### 4.8. Comparison with Similar Work

Numerical values achieved by the proposed solution were compared with values reported in several papers dealing with similar plaintext-related image encryption algorithms. Older approaches use simpler plaintext-related stages that may obtain insufficient values of some numerical parameters [25,27] or whole algorithms may be already broken [30]. Some of the newer proposals utilize complex chaotic systems and do not focus on the fine-tuning of their performance [32,33,34,35,37]. The values of numerical parameters obtained for the red color plane of true color image lena or grayscale image lenaG with resolutions of 512 × 512 pixels are included in Table 7.

The proposed solution achieves the best values of correlation coefficients ρ and entropy *H* among algorithms reporting results for the red color plane of true color image lena. Values of NPCR and UACI are comparable with the best approach [25] in this category. The number of processor cycles necessary for the encryption of one byte $cyc_{enc}$ is higher than that achieved by [30]; however, this scheme was broken [31].

A comparison of algorithms that report numerical values for grayscale image lena shows that the values of ρ achieved by the proposed solution are close to the best values obtained by [35]. However, the proposed algorithm has much more balanced results. The highest value of *H* is achieved by [37], closely followed by the proposed algorithm and almost all other solutions. Obtained values of NPCR and UACI are also among the best as the proposed algorithm ranks second behind [35] for NPCR and third behind [33,35] for UACI. The most significant advantage of the proposed solution is its computational complexity, which is by far the lowest. The second-fastest algorithm [19] is almost three-times slower.

The reported numerical parameters show that the proposed solution is able to reach values of numerical parameters that are comparable with those achieved by more complex approaches that use either hash functions [32,33,34,35] or special coding techniques [18,37]. As our proposal is the fastest one, and considering that the presented values of numerical parameters are close to the best, the proposed algorithm can be viewed as effective and our initial assumptions about its properties are proven to be correct.

## 5. Conclusions

This paper dealt with the topic of plaintext-related image encryption algorithms. After a brief introduction to the area and a survey of relevant approaches, a novel solution was proposed. It merges the latest knowledge about the LM and its properties from our previous work, a new plaintext-related parameter modification technique and a rather uncommon row-wise approach of pixel intensity processing that enables the extraction of all required values during both encryption and decryption. Experimental results showed that the proposed algorithm is sensitive even to small differences between various plain images or keys and the sequence modified according to the plain image pixel intensities passed all statistical tests from the NIST 800-22 test suite. The numerical results were compared with those reported in similar work and it was found that, although our proposal uses a simpler chaotic map, it can obtain the same results as algorithms utilizing complex chaotic systems. Furthermore, the computational complexity of the proposed scheme is much lower that that of other algorithms.

The presented results confirm an idea from our previous research—finely tuned, simpler chaotic systems can manifest similar behavior to some inappropriate implementations of more complex chaotic systems. Moreover, the simpler chaotic maps were popularized much sooner and they have been analyzed more precisely. In our future work, we would like to investigate other methods of utilizing the full potential of simple chaotic maps such as the LM in the area of plaintext-related image encryption. These may include modifications of Equation (1) for the enhancement of its chaotic behavior, the usage of different quantization techniques that may obtain multiple sequence elements from one iterate or more efficient parameter value patterns.

## Figures and Tables

**Table 1:** Key schedule, sequence lengths and maximal element values for all generated sequences.

Generated Sequence	Parameter Values Pattern								Sequence Length	Maximal Element Value *Q*
$seq_{1}$	$r_{4}$	$r_{8}$	$r_{3}$	$r_{7}$	$r_{2}$	$r_{6}$	$r_{1}$	$r_{5}$	$w^{\prime }$	h−1
$seq_{2}$	$r_{5}$	$r_{1}$	$r_{6}$	$r_{2}$	$r_{7}$	$r_{3}$	$r_{8}$	$r_{4}$	*h*	$w^{\prime }-1$
$seq_{3}$	$r_{1}$	$r_{2}$	$r_{3}$	$r_{4}$	$r_{5}$	$r_{6}$	$r_{7}$	$r_{8}$	$h\times w^{\prime }$	255
$seq_{4}$	$r_{2}$	$r_{1}$	$r_{4}$	$r_{3}$	$r_{6}$	$r_{5}$	$r_{8}$	$r_{7}$	$w^{\prime }$	h−1
$seq_{5}$	$r_{7}$	$r_{8}$	$r_{5}$	$r_{6}$	$r_{3}$	$r_{4}$	$r_{1}$	$r_{2}$	*h*	$w^{\prime }-1$
$seq_{6}$	$r_{8}$	$r_{7}$	$r_{6}$	$r_{5}$	$r_{4}$	$r_{3}$	$r_{2}$	$r_{1}$	$num_{px}$	255

**Table 2:** Combinations used during four scanning directions.

Scanning Direction	Scanning Order		Addition (Mod 256)	XOR
	Rows *l*	Columns *k*		
top to bottom	1,2,3,⋯,h	:	l−1	l+1
left to right	:	$1,2,3,\cdots ,w^{\prime }$	k−1	k+1
bottom to top	h,h−1,h−2,⋯,1	:	l+1	l−1
right to left	:	$w^{\prime },w^{\prime }-1,w^{\prime }-2,\cdots ,1$	k+1	k−1

Note: A colon : stands for all possible row or column indexes.

**Table 3:** A set of experimental keys.

Key	Value
$K_{1}$	0× C9 0F DA A2 21 68 C2 34 C4 C6 62 8B 80 DC 1C D1
$K_{2}$	0× C9 0F DA A2 21 68 C2 34 C5 C6 62 8B 80 DC 1C D1
$K_{3}$	0× 00 00 00 00 00 00 00 00 00 00 00 00 00 00 00 00

**Table 4:** Results of the tests from NIST 800-22 suite obtained by the plaintext-related sequence.

Test	Required Pass Rate	Obtained Successful Results
Frequency (monobit)	96/100	99/100
Frequency within a block (*M* = 128 bits)	96/100	98/100
Runs	96/100	96/100
Longest run of ones in a block	96/100	97/100
Binary matrix rank	96/100	98/100
Discrete Fourier transform (spectral)	96/100	98/100
Non-overlapping template matching (*m* = 9 bits, first *p*-value)	96/100	97/100
Overlapping template matching (*m* = 9 bits)	96/100	96/100
Maurer’s universal statistic	96/100	98/100
Linear complexity (*M* = 500 bits)	96/100	98/100
Serial (*m* = 16 bits, first *p*-value)	96/100	98/100
Approximate entropy (*m* = 10 bits)	96/100	96/100
Cumulative sums (first *p*-value)	96/100	99/100
Random excursions (first *p*-value)	60/63	62/63
Random excursions variant (first *p*-value)	60/63	63/63

**Table 5:** Achieved values of numerical parameters.

Image, Color Plane and Key		var [-]	$\rho_{h}$ [-]	$\rho_{v}$ [-]	$\rho_{d}$ [-]	*H* [bits/px]	NPCR [%]	UACI [%]
lena	R	510,371	0.9723	0.9731	0.9535	7.5889	not reported	
	G	1,290,286	0.9734	0.9709	0.9531	7.106		
	B	1,908,534	0.9702	0.9733	0.9528	6.8147		
$K_{1}$	R	1094	−0.0019	0.002	−0.0012	7.9992	99.6143	33.4857
	G	1003	−0.0005	−0.0018	−0.0001	7.9993	99.6134	33.4805
	B	929	−0.0014	−0.001	−0.0022	7.9994	99.6144	33.4811
$K_{2}$	R	1064	0.0017	0.0008	−0.0016	7.9993	99.6135	33.4816
	G	1046	−0.001	0.0024	0.0021	7.9993	99.6142	33.4839
	B	1137	0.0002	0.0004	−0.0014	7.9992	99.6145	33.4822
$K_{3}$	R	1024	−0.0029	0.0017	0.0011	7.9993	99.6151	33.486
	G	1007	−0.0015	0.0012	−0.0018	7.9993	99.6138	33.4823
	B	909	−0.0027	−0.0003	0.0007	7.9994	99.6157	33.4855
peppers	R	852,749	0.9577	0.965	0.9477	7.3388	not reported	
	G	1,273,532	0.9609	0.9681	0.9558	7.4963		
	B	1,965,713	0.963	0.965	0.9523	7.0583		
$K_{1}$	R	1012	0.0024	−0.0004	−0.0013	7.9993	99.614	33.4816
	G	988	−0.0006	0.0017	0.0012	7.9993	99.6164	33.4832
	B	1099	−0.0003	0.0028	−0.001	7.9992	99.6154	33.4818
$K_{2}$	R	878	0.0006	0.0014	−0.0023	7.9994	99.6146	33.4818
	G	909	0.0014	−0.0005	0.0007	7.9994	99.6132	33.4808
	B	841	−0.0007	−0.001	0.0025	7.9994	99.6166	33.4846
$K_{3}$	R	1070	0.0017	0.0022	−0.0004	7.9993	99.6158	33.4864
	G	947	0.0011	0.001	−0.0009	7.9993	99.6157	33.4874
	B	1036	−0.0006	−0.0003	0.0023	7.9993	99.6147	33.483
baboonG	-	750,764	0.8435	0.7129	0.6758	7.3579	not reported	
$K_{1}$		1123	0.0013	0.0014	0.0001	7.9992	99.6153	33.4807
$K_{2}$		1102	−0.0009	−0.0027	0.0014	7.9992	99.6142	33.4808
$K_{3}$		1046	−0.0019	−0.0013	−0.0004	7.9993	99.6149	33.4856
lenaG	-	1,039,126	0.9709	0.9765	0.9561	7.2344	not reported	
$K_{1}$		993	0.0006	−0.0015	−0.0008	7.9993	99.6127	33.483
$K_{2}$		1046	−0.0009	0.0029	0.0022	7.9993	99.6138	33.4836
$K_{3}$		995	−0.0013	0.0026	0.0018	7.9993	99.6144	33.4847
peppersG	-	478,900	0.9698	0.9767	0.9628	7.5943	not reported	
$K_{1}$		1108	0.0013	−0.0006	−0.0012	7.9992	99.614	33.484
$K_{2}$		1075	−0.002	−0.0007	−0.0016	7.9993	99.6132	33.4847
$K_{3}$		935	0.0007	−0.0026	0.0004	7.9994	99.6137	33.4806
sailboatG	-	718,875	0.9748	0.9657	0.9538	7.4847	not reported	
$K_{1}$		918	0.0011	−0.0004	−0.0016	7.9994	99.6135	33.4844
$K_{2}$		1042	−0.0003	−0.002	0.0014	7.9993	99.6154	33.4824
$K_{3}$		906	0.0019	−0.0019	−0.0003	7.9994	99.6148	33.4855

Note: A dash—means that plain image is grayscale.

**Table 6:** Measured and computed values of computational complexity.

Image and Key		$t_{\mathrm{enc}}$ [ms]	$t_{\mathrm{dec}}$ [ms]	$v_{\mathrm{enc}}$ [MB/s]	$v_{\mathrm{dec}}$ [MB/s]	${\mathrm{cyc}}_{\mathrm{enc}}$ [cycles/B]	${\mathrm{cyc}}_{\mathrm{dec}}$ [cycles/B]
lena	$K_{1}$	493.0626	487.9469	1.5211	1.5371	1567.4	1551.14
	$K_{2}$	490.6985	488.0388	1.5284	1.5368	1559.89	1551.43
	$K_{3}$	493.014	487.4362	1.5211	1.5371	1567.25	1549.52
peppers	$K_{1}$	491.7683	488.2473	1.5251	1.5361	1563.29	1552.1
	$K_{2}$	492.1848	490.5268	1.5238	1.529	1564.61	1559.34
	$K_{3}$	491.2771	488.7079	1.5266	1.5347	1561.73	1553.56
baboonG	$K_{1}$	149.4431	143.812	1.6729	1.7384	1425.2	1371.5
	$K_{2}$	149.4405	143.4985	1.6729	1.7422	1425.18	1368.51
	$K_{3}$	149.2358	143.409	1.6752	1.7433	1423.22	1367.65
lenaG	$K_{1}$	149.4753	143.4511	1.6725	1.7428	1425.51	1368.06
	$K_{2}$	149.3001	143.7227	1.6745	1.7395	1423.84	1370.65
	$K_{3}$	149.5298	144.3434	1.6719	1.732	1426.03	1376.52
peppersG	$K_{1}$	149.5122	143.768	1.6721	1.7389	1425.86	1371.08
	$K_{2}$	149.4405	143.4985	1.6729	1.7422	1425.18	1368.51
	$K_{3}$	149.2358	143.409	1.6752	1.7433	1423.22	1367.65
sailboatG	$K_{1}$	149.2376	143.7204	1.6752	1.7395	1423.24	1370.62
	$K_{2}$	149.1357	143.3698	1.6763	1.7437	1422.27	1367.28
	$K_{3}$	149.1004	143.4411	1.6767	1.7429	1421.93	1367.96

**Table 7:** Comparison of obtained numerical results with similar work.

Approach	$\rho_{h}$ [-]	$\rho_{v}$ [-]	$\rho_{d}$ [-]	*H* [bits/px]	NPCR [%]	UACI [%]	${\mathrm{cyc}}_{\mathrm{enc}}$ [cycles/B]
Red color plane of true color image lena							
proposed	−0.0019	0.002	−0.0012	7.9992	99.6143	33.4857	1567.4
[25]	−0.0029	−0.015	0.0129	7.997	99.62	33.51	∼2270
[30]	0.0135	-		7.9974	99.63	33.31	648.53
Grayscale image lenaG							
proposed	0.0006	−0.0015	−0.0008	7.9993	99.6127	33.483	1425.51
[19]	0.0077	0.0053	0.0003	7.9993	99.606	33.4714	4205.32
[27]	−0.0046	−0.0511	−0.0168	7.9993	99.6101	33.4679	8230.32
[32]	0.0044	0.0151	0.0012	7.9993	99.62	33.45	15,120.97
[33]	−0.0037	−0.0029	0.0047	7.9975	99.5956	33.5512	43,151.97
[34]	0.0013	0.0008	0.0066	7.9993	99.6107	33.436	5185.19
[35]	0.0003	0.0019	0.0003	7.9993	99.6159	33.4846	4945.37
[37]	−0.0003	−0.0024	−0.0022	7.9994	99.6096	33.4599	72,452.57

Note: A dash-stands for non-reported data.

## Data Availability

The data presented in this study are available on request from the corresponding author.

## Abbreviations

The following abbreviations are used in this manuscript:

AES	Advanced Encryption Standard
CPU	Central Processing Unit
ECB	Electronic CodeBook
LE	Lyapunov Exponent
LM	Logistic Map
NPCR	Number of Pixel Change Ratio
OS	Operating System
RAM	Random Access Memory
UACI	Unified Average Changing Intensity
XOR	eXclusive OR
